# Pharmacological blockade of dopamine D1- or D2-receptor in the prefrontal cortex induces attentional impairment in the object-based attention test through different neuronal circuits in mice

**DOI:** 10.1186/s13041-021-00760-3

**Published:** 2021-02-28

**Authors:** Bolati Wulaer, Kazuo Kunisawa, Moeka Tanabe, Aika Yanagawa, Kuniaki Saito, Akihiro Mouri, Toshitaka Nabeshima

**Affiliations:** 1 Advanced Diagnostic System Research Laboratory, Aichi, Japan; 2 Department of Disease Control and Prevention, Aichi, Japan; 3 Department of Regulatory Science for Evaluation & Development of Pharmaceuticals & Devices, Fujita Health University Graduate School of Health Science, 1-98 Dengakugakubo, Kutsukake-cho, Toyoake, Aichi 470-192 Japan; 4 Japanese Drug Organization of Appropriate Use and Research, Nagoya, Aichi Japan

**Keywords:** Attention, OBAT, Prefrontal cortex, Dopamine, D1 receptor, D2 receptor, Antagonist, C-Fos

## Abstract

Dopamine is a key neurotransmitter that regulates attention through dopamine D1 and D2-receptors in the prefrontal cortex (PFC). We previously developed an object-based attention test (OBAT) to evaluate attention in mice. Disruption of the dopaminergic neuronal system in the PFC induced attentional impairment in the OBAT. However, previous studies have not systematically examined which specific brain regions are associated with the blockade of PFC dopamine D1 and D2-receptors in the OBAT. In this study, we investigated the association of dopamine D1 and D2-receptors in the PFC with attention and neuronal activity in diverse brain regions. We found that both dopamine D1 and D2-receptor antagonists induced attentional impairment in the OBAT by bilateral microinjection into the PFC of mice, suggesting that both dopamine D1 and D2-receptors were associated with attention in the OBAT. Our analysis of the neuronal activity as indicated by c-Fos expression in 11 different brain regions showed that based on the antagonist types, there was selective activation of several brain regions. Overall, this study suggests that both dopamine D1 and D2-receptors play a role in attention through different neuronal circuits in the PFC of mice.

## Main text

Attention is critical to high-level cognition, while attention deficit is a hallmark of neuropsychiatric disorders [1]. The dopaminergic neuronal system is considered a primary pharmacological target for such disorders and it also contributes to attention [2]. Dopamine receptors are G-protein-coupled receptors and are classified into two families: excitatory D1-class receptors (D1 and D5) and inhibitory D2-class receptors (D2, D3, D4) [3]. Within these families, dopamine D1 and D2-receptors are the most abundant receptor subtypes in the brain that are characterized both pharmacologically and physiologically. These are pre-synaptically localized in nerve terminals and axonal varicosities as well as post-synaptically localized in dendritic shafts and spines [4].

In an earlier study, we established an object-based attention test (OBAT) to evaluate attention in mice [5]. This test was based on the inherent behavior of mice, which was of exploration of novelty without external reinforcers or any instrumental training. There is evidence that attentional impairment in the OBAT is associated with the disruption of the dopaminergic neuronal system, such as the reduction of dopamine transporter and dopamine D5-receptor deficiency in mice [6, 7]. The prefrontal cortex (PFC) is a major forebrain dopamine target that mediates attentional function [8]. Furthermore, our previous study confirmed that the PFC is necessary for attention in OBAT [9]. In a separate study, we found that attentional impairment in OBAT was associated with reduced neuronal activity in the PFC of a mouse model with dopaminergic dysfunction (manuscript in preparation). However, previous studies have not systematically examined which specific brain regions are associated with dopamine D1 and D2-receptors in the PFC in the OBAT. Therefore, in this study, the functional contribution of dopamine D1 and D2-receptors to attention and neuronal activity in the PFC was analyzed in diverse brain regions in mice.

**Fig. 1:**
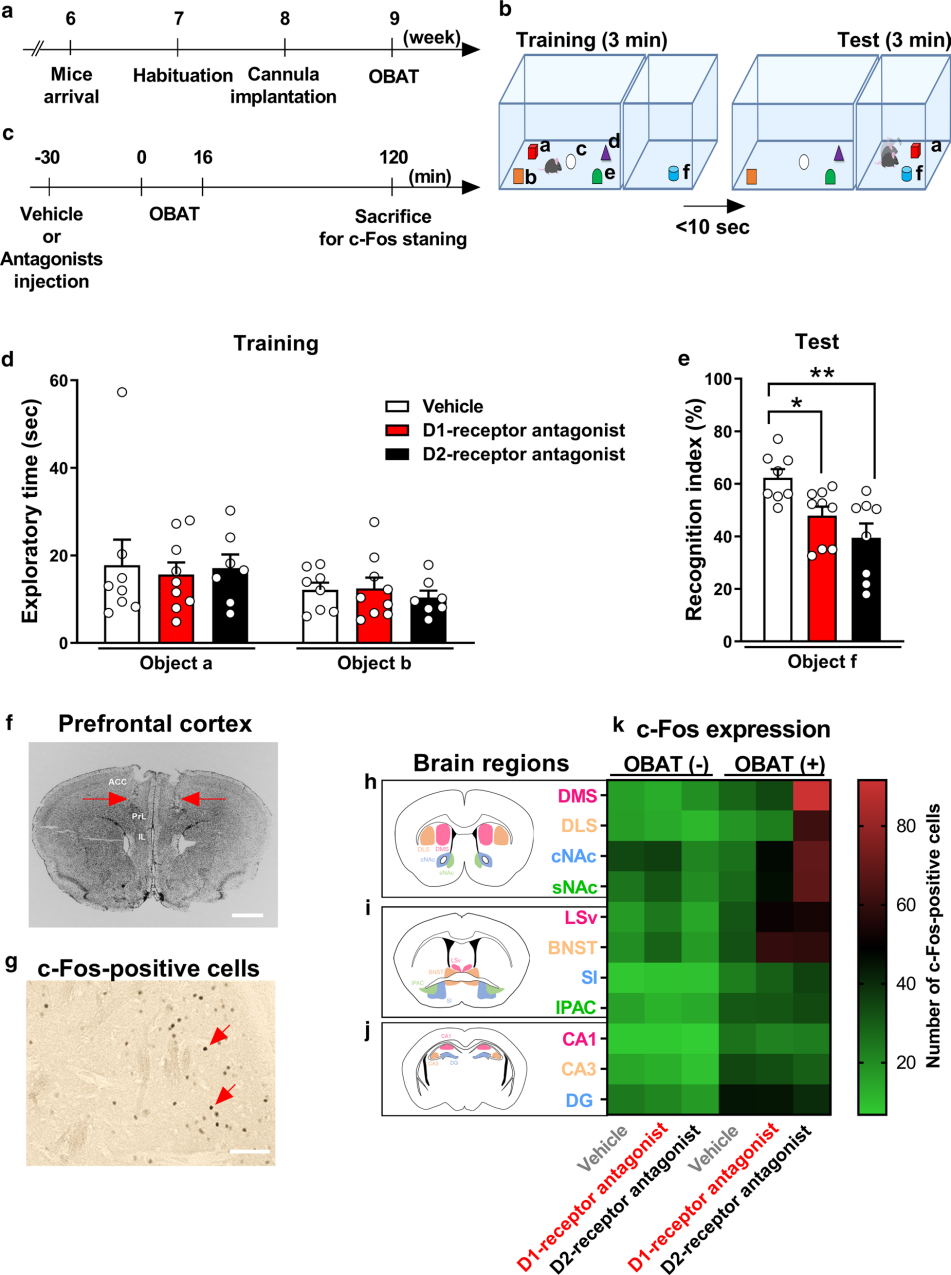
Dopamine D1- and D2-receptor antagonists impair attention in the OBAT by injection into the PFC of mice. **a** Experimental protocol of the entire procedures. **b** Protocol for OBAT. **c** Experimental protocol of OBAT and c-Fos staining. **d** The exploratory time in the training and **e** recognition index in the testing of OBAT (vehicle = 8 mice, D1-receptor antagonist = 9 mice, D2-receptor antagonist = 8 mice). **f** Representative image of the injection site (scale bar: 1000 µm). **g** Representative image of the c-Fos-positive cells (scale bar: 100 µm). **h** Schematic brain area of DMS, DLS, cNAc, sNAc, **i** LSv, BNST, IPAC, SI, **j** CA1, CA3 and DG referred from the Allen Mouse Brain Atlas. **k** The c-Fos expression in the heatmap form. * *p* < 0.05, ** *p* < 0.01. All data are expressed as means ± SEM. PFC, prefrontal cortex; OBAT, object-based attention test; anterior cingulate cortex (ACC); prelimbic cortex (PrL); infralimbic cortex (IL); dorsomedial striatum (DMS); dorsolateral striatum (DLS); nucleus accumbens core (cNAc), nucleus accumbens shell (sNAc); lateral septal nucleus ventral part (LSv); bed nucleus of the stria terminalis (BNST); interstitial nucleus of the posterior limb of the anterior commissure (IPAC); substantia innominate (SI); cornu ammonis 1 (CA1); cornu ammonis 3 (CA3); dentate gyrus (DG)

Six-week-old mice were habituated for a 1-week period in an animal facility (Fig. 1a). Regular handling was performed to reduce stress and anxiety in mice 1 week after arrival (5 min/day for 3 days). Then, guide cannulas were inserted into the PFC of the mice. The mice were given another week to fully recover from the surgery before the OBAT. In the OBAT, mice were introduced to a training (familiarization) session, where they were presented with five (Fig. 1b, a–e) differently shaped but similarly sized objects for 3 min, within an interval of less than 10 s; a novel (e.g., f) and familiar (e.g., a) object was then presented during testing for another 3 min (Fig. 1b). On the testing day, vehicle or antagonists were injected through the guide cannulas 30 min before the test started, and the brain samples were collected 120 min after the start of the test (Fig. 1c). No changes were observed in any group during the training (two-way ANOVA, groups, (F_2, 42_) = 0.0686, *p* = 0.9337; objects, (F_1, 42_) = 3.7780, *p* = 0.0586; groups × objects interaction, (F_2, 42_) = 0.1640, *p* = 0.8493; Fig. 1d). In the testing, mice showed a decreased recognition index after injection of either dopamine D1 or D2 receptor antagonist into the PFC, suggesting impaired attention (one-way ANOVA, (F_2, 22_) = 0.2772, *p* = 0.0032, Fig. 1e). The injection site is shown in Fig. 1f. Taken together, these results indicate that blockade of both dopamine D1 and D2-receptors in the PFC induces attentional impairment in the OBAT, thus indicating the involvement of the dopamine D1 and D2-receptors in the PFC. These results are consistent with those of previous studies, which show that disturbance of dopamine receptors results in attentional impairment in rodents [10].

Figure 1g shows a typical image of c-Fos-positive cells. Our analysis of the neuronal activity as indicated by c-Fos expression in 11 different brain regions showed that attentional impairment was accompanied by the induction of c-Fos expression in multiple brain regions after the OBAT. The blockade of the dopamine D1-receptor in the PFC specifically induced c-Fos expression in the nucleus accumbens core (cNAc), lateral septal nucleus ventral part (LSv), and bed nucleus of the stria terminalis (BNST). Alternatively, the blockade of dopamine D2-receptor in the PFC induced c-Fos expression in the dorsomedial striatum (DMS), dorsolateral striatum (DLS), cNAc, nucleus accumbens shell (sNAc), LSv, and BNST (See Fig. 1h–k, Additional file 1: Table S1 for numeric values). Using task-based functional MRI, human patients with attention-deficit/hyperactivity disorder (ADHD) also show reduced functional connectivity among the PFC, nucleus accumbens (NAc), and striatum [11]. The regional differences in c-Fos expression by the blockade of dopamine D1 and D2-receptors suggest that these dopamine receptors may play different roles in the attentional neuronal circuit. ADHD is associated with several forms of risk-taking behavior. NAc receives projections from the PFC, disruption of modulation by dopamine D1-receptor in PFC-NAc pathway reduces risky choice [12]. In this study, we showed the blockade of dopamine D1-receptor in the PFC induced c-Fos expression in the cNAc and the blockade of dopamine D2-receptor in the PFC induced c-Fos expression in the cNAc and sNAc. These data suggested that cNAc and/or sNAc might contribute risk-taking behavior via attention deficit induced by the blockade of dopamine D1 and D2-receptors. Neurons expressing dopamine D1 and D2-receptor in the PFC is different but both neurons project striatum [13, 14]. Corticostriatal circuit is important for the goal-directed action [15], which contribute attention [16]. In this study we showed that blockade of dopamine D2-receptor in the PFC induced c-Fos expression in the DMS and DLS. These data suggested that DMS and DLS might contribute attention deficit induced by the blockade of dopamine D2- receptors which modulates goal-directed action. Both antagonists failed to induce c-Fos expression in the CA1, CA3, and DG of the hippocampus after OBAT, but we noted a significantly increased expression of c-Fos expression in all the OBAT-exposed groups as compared to the behavior naïve condition (arena control), suggesting that the hippocampus itself is necessary for the attention of OBAT. This finding was similar to that of a previous report that mentioned the hippocampus being implicated in the regulation of attention [9]. The hippocampus-PFC circuit is implicated in attention [17]. Surprisingly, both dopamine antagonists failed to affect c-fos expression in the hippocampus subregions. These data suggest that contribution of PFC dopamine is limited in the hippocampus-PFC circuit for attention in the OBAT. The c-Fos expressions induced by dopamine D1 and D2-receptor antagonists are observed only after the OBAT but not without the OBAT. This is probably partially due to the neural circuits (or neuronal activation) associated with dopamine D1 and D2-receptors is important to enhance the c-fos expression with OBAT performance.

Our study is limited because the exact role of the brain area being manipulated can be obscured by pharmacological suppression [18]. Dopamine D1 and D2 receptors in the PFC are selectively expressed in different classes of frontal output neurons; dopamine D1 receptors are mainly interneurons, whereas the dopamine D2 receptors are both small pyramidal cells and interneurons. Thus, they might be able to influence different neural circuit in the brain [13]. The details how inhibition of both dopamine D1 and D2-receptors activated the brain regions outside of PFC are remain largely unknown. Dopamine D1-receptor inhibition might affect the postsynaptic currents in pyramidal cells, or alter non- N-methyl-D-aspartate glutamatergic responses; dopamine D2-receptor inhibition might change inhibitory currents in pyramidal cells as well as the interneuron excitability, leading to an imbalance between excitation and inhibition; thereby they alter the neuronal activity in the existing neuronal circuit [19]]. Therefore, it is important to have a more precise temporal control of brain circuit manipulation so that neuronal activity can be manipulated over brief periods of time corresponding to the training, testing, or both in the OBAT. The future research should investigate: (1) identification of neuronal subgroups that selectively project to the region of interest with retrograde virus combined with Cre driver mouse lines; and (2) specific neuronal manipulation within a specific neuronal projection in a temporally precise manner in behaving animals, thereby allowing assessment of the precise contribution of a given neural population in the brain region to attentional behavior. Despite this limitation, this is the first study to identify attentional impairment induced by dopamine D1 and D2-receptor antagonists injected into the PFC, which may play different roles in the attentional neural circuit of OBAT. Overall, this study provides hints to identify neural circuits that underlie the attention response of the PFC dopamine D1 and/or D2-receptor in mice.

## Supplementary Information

**Additional file 1.** Additional materials.

## Data Availability

All data used in this study are available from the corresponding author upon reasonable request.

## Abbreviations

ACC: Anterior cingulate cortex
ADHD: Attention-deficit/hyperactivity disorder
BNST: Analysis of variance (ANOVA), bed nucleus of the stria terminalis
CA: Cornu ammonis
DG: Dentate gyrus
DLS: Dorsolateral striatum
DMS: Dorsomedial striatum
IPAC: Interstitial nucleus of the posterior limb of the anterior commissure
IL: Infralimbic cortex
LSv: Lateral septal nucleus ventral part
cNAc: Nucleus accumbens core
sNAc: Nucleus accumbens shell
OBAT: Object-based attention test
PFC: Prefrontal cortex
PrL: Prelimbic cortex
SI: Substantia innominate
