# Supplementary material for: Pharmacological blockade of dopamine D1- or D2-receptor in the prefrontal cortex induces attentional impairment in the object-based attention test through different neuronal circuits in mice
Source: Mol Brain. 2021 Feb 28;14:43. doi: 10.1186/s13041-021-00760-3 (PMC7916264; doi:10.1186/s13041-021-00760-3)
Supplement: Supplementary file 1 — Additional file 1. Additional materials. [file 13041_2021_760_MOESM1_ESM.docx]

**Additional file**

**Pharmacological blockade of dopamine D1 or D2-receptor in the prefrontal cortex induces attentional impairment in the object-based attention test through different neuronal circuits in mice**

Bolati Wulaer, Kazuo Kunisawa, Moeka Tanabe, Aika Yanagawa, Kuniaki Saito, Akihiro Mouri and Toshitaka Nabeshima

**Materials and Methods**

**Animals**

Male C57BL/6J mice (6 weeks old) were purchased from Japan SLC Inc. (Shizuoka, Japan). Male mice were used to avoid potential estrus cycle-related performance variability in female mice (1). The sample sizes were based on previous studies (2, 3). Animals were housed in the animal facilities at the Fujita Health University Graduate School of Medicine under specific pathogen-free conditions, maintained at 25°C on a 12-h light/dark cycle (lights on at 08:00), and they had free access to food and water. The animals were acclimatized for 1 week prior to the start of the experiments. All animal care and use were performed in accordance with the National Institute of Health Guide for the Care and Use of Laboratory Animals and was approved by the Animal Experimentation Committee of Fujita Health University Graduate School of Medicine (Permit Number: APU19119).

**Mouse stereotaxic surgery**

Mice were anesthetized with a mixture of anesthetic, muscle relaxant, analgesic, and sedative such as medetomidine (0.3 mg/kg; Domitor®; Nippon Zenyaku Kogyo), butorphanol (5.0 mg/kg; Vetorphale®; Meiji Seika Pharma), midazolam (4.0 mg/kg; Midazolam Sandoz®; Sandoz) to minimize the pain and reversed by atipamezole (0.15 mg/kg; Antisedan®; Nippon Zenyaku Kogyo) after completion of the surgery. Stainless steel guide cannulas (9.7 mm, 0.4 mm inner diameter, 0.5 mm outer diameter; Cat# AG-6; Eicom) were bilaterally implanted into the prefrontal cortex (AP=+1.54, ML=±0.50, DV=−2.00) (4). The guide cannulas were fixed using dental cement (Cat# 204310196; Shofu Inc.). To seal the top of the guide cannula and prevent tissue entry into the cannula, a dummy cannula (0.3 mm in diameter; Cat# AD-6; Eicom) was left in place throughout the experiment. On the testing day, mice were injected with vehicle, dopamine D1-receptor antagonist (SCH23390; 1 μg/0.5 μL/side; Cat# D054; Sigma-Aldrich), or dopamine D2-receptor antagonist (Raclopride; 5 μg/0.5 μL/side; Cat# 84225-95-6; Tokyo Chemical Industry Co. Ltd.) 30 min before the start of the OBAT using a 27-gauge infusion needle (1.0 mm longer than the guide cannulas; Cat# 80300; Plastics One) connected to a 10 μl Hamilton microsyringe at a rate of 0.1 μl/min. SCH23390 and raclopride were dissolved in saline. The protocol was chosen on the basis of a previous study in which the dose induced cognitive impairment in the novel object recognition test (5). Based on the evidence in a previous study, the local administration of D1- or D2-receptor antagonists in the PFC does not affect locomotor activity (6).

**Object-based attention test (OBAT)**

We established an OBAT in our previous study (7). A rectangular, two-chambered, opaque plexiglass box including an exploring chamber (40 cm × 40 cm × 22 cm) and test chamber (40 cm × 20 cm × 22 cm) was used for this study. The dividing walls were made of opaque plexiglass with sliding openings that allowed access between each chamber. Briefly, mice were allowed to explore both the training and testing chambers for a total of 10 min during the habituation period. In the training session, mice were exposed to five similar-sized but differently shaped objects (e.g., objects a-e in Figure 1b) for 3 min. A previous study reported that a mouse spent an equal amount of time exploring the five objects during a 3-min exposure (7). Therefore, the time spent exploring two randomly selected objects out of the five was recorded. Mice were immediately (less than 10 s) moved to the test session where familiar (e.g., object a) and novel (e.g., object f) objects were introduced accordingly. The recognition index was expressed as the ratio (Tf × 100)/(Ta + Tf), where Ta and Tf were the times spent in the testing session exploring objects a and f, respectively (Figure 1b).

**3, 3’-diaminobenzidine (DAB) staining**

DAB staining was performed as described previously (2). Two hours after the start of the OBAT, brains were removed transcardially, post-fixed overnight in 4% paraformaldehyde, and cryoprotected in 30% sucrose in phosphate-buffered saline (PBS). Brain tissues were prepared (30 μm) using a cryostat (RRID:SCR_016844; Leica CM3050 S Research Cryostat, Germany). The coronal sections were prepared, washed with PBS containing 0.3% Triton X-100, and incubated at room temperature (~ 20–25°C) for 2 h in the presence of 2% normal goat serum (RRID: AB_2336820; Cat# S-1000; Vector Laboratories Inc., USA), and then incubated with a rabbit anti-c-Fos antibody (RRID: AB_2231974; Cat# 226003; Synaptic Systems; Germany; 1:1000) at 4°C overnight. After being washed with PBS, the sections were incubated with a biotinylated goat anti-rabbit IgG secondary antibody (RRID: AB_2336820; Cat# BA-1000; Vector Laboratories Inc., 1: 1,000) at room temperature for 2 h. Then, the sections were incubated with PBS containing 0.3% hydrogen peroxide (#08104215; FUJIFILM Wako Pure Chemical Corporation, Japan) for 30 min to inactivate endogenous peroxidase. Thereafter, the sections were washed with PBS and incubated with the avidin-conjugated horseradish peroxidase complex (RRID: AB_2336820; Cat# PK-6100; Vectastain ABC kit, Vector Laboratories Inc.) at room temperature for 2 h. The signal was visualized using the diaminobenzidine–nickel staining method (2). The sections were stained using 1 mg/mL DAB (#D5637; Sigma-Aldrich), 30% hydrogen peroxide, and 4% nickel chloride (Cat# 149–05343; FUJIFILM Wako Pure Chemical Corporation). c-Fos expression was quantified by counting the number of c-Fos-positive cells in the region of interest. Only those cells that had significant above background levels of DAB staining in their nuclei were counted. The acquisition parameters were the same for all images. The number of c-Fos-positive cells was counted within an area of 340 µm × 260 µm using ImageJ software (RRID:SCR_003070; National Institute of Mental Health, USA). The average of three slices in each mouse was calculated and used for statistical analysis. The selection of these brain regions was based on previous studies that focused on forebrain regions involved in cognition with slight changes only (4, 8).

**Data analyses**

Statistical analyses were performed using GraphPad Prism 6.0 (GraphPad Software, Inc., CA, USA). All data were expressed as the mean ± SEM. One-or two-way ANOVA tests followed by Tukey’s post-hoc test were used for statistical analyses. All mice were randomly divided into the experimental groups. No data points were excluded from any of the experiments unless the mice died during or after the stereotaxic procedure. The criterion for a significant difference was **p* < 0.05, and ***p* < 0.01, in all statistical evaluations.

**Additional file Table 1: Changes in c-Fos expression in 11 different brain regions by injection of dopamine D1- or D2-receptor antagonist into prefrontal cortex**

| Brain region | OBAT (-) | | | OBAT (+) | | |
| --- | --- | --- | --- | --- | --- | --- |
|  | Vehicle | D1-RA | D2-RA | Vehicle | D1-RA | D2-RA |
| DMS | 16.33±2.12 | 13.61±0.51 | 19.56±4.01 | 28.17±2.71 | 34.28±2.02 | 91.61±18.07^$$,&&^ |
| DLS | 16.44±1.79 | 14.11±1.37 | 11.17±2.05 | 18.17±2.86 | 23.06±3.23 | 61.78±17.57^$$,&&^ |
| cNAc | 34.33±5.34 | 36.28±3.72 | 21.67±2.39 | 26.00±2.46 | 47.50±5.24^&^ | 69.17±7.28^$$,&&^ |
| sNAc | 25.17±2.81 | 31.61±4.23 | 20.28±1.12 | 27.78±2.51 | 46.17±4.18 | 68.56±8.45^$$,&&^ |
| LSv | 17.27±3.36 | 24.28±1.85 | 14.67±2.22 | 31.78±4.65 | 51.28±5.56^&&, ##^ | 53.39±3.07^$$,&&^ |
| BNST | 19.87±2.15 | 28.94±2.92 | 17.17±1.73 | 32.61±6.01 | 59.67±3.69^&&, ##^ | 58.94±8.58^$$,&&^ |
| IPAC | 15.67±2.90 | 12.22±0.99 | 14.06±1.68 | 31.22±5.42 | 31.50±3.52 | 34.11±8.96 |
| SI | 8.00±0.37 | 8.94±0.92 | 9.11±0.79 | 23.06±1.25 | 29.61±4.17^#^ | 35.44±8.34^$$^ |
| CA1 | 7.83±1.27 | 7.72±0.98 | 6.83±0.94 | 25.56±4.60** | 22.44±1.79^##^ | 23.33±1.85^$$^ |
| CA3 | 14.67±1.05 | 12.78±0.62 | 9.56±1.74 | 34.56±3.13** | 33.11±2.17^##^ | 29.39±1.52^$$^ |
| DG | 24.11±2.02 | 21.28±2.59 | 17.11±2.44 | 44.83±3.41** | 44.50±2.37^##^ | 39.94±2.94^$$^ |

Data are expressed as mean ±SEM c-fos-positive cells. n = 6 each group. See Figure 1k for the summarized data in heatmap form. ** *p* < 0.01 vs. OBAT (-) vehicle; ^#^*p* < 0.05, ^##^*p* < 0.01 vs. OBAT (-) D1-receptor antagonist; ^$$^*p* < 0.01 vs. OBAT (-) D2-receptor antagonist; ^&^*p* < 0.05 vs. OBAT (+) vehicle; ^&&^ *p* < 0.01 vs. OBAT (+) vehicle. OBAT, object-based attention test; D1-RA, D1-receptor antagonist; D2-RA, D2-receptor antagonist; dorsomedial striatum (DMS); dorsolateral striatum (DLS); nucleus accumbens core (cNAc), nucleus accumbens shell (sNAc), lateral septal nucleus ventral part (LSv), bed nucleus of the stria terminalis (BNST), interstitial nucleus of the posterior limb of the anterior commissure (IPAC), substantia innominate (SI), cornu ammonis 1 (CA1), cornu ammonis 3 (CA3), dentate gyrus (DG).

**References**

1. Meziane H, Ouagazzal AM, Aubert L, Wietrzych M, Krezel W. Estrous cycle effects on behavior of C57BL/6J and BALB/cByJ female mice: implications for phenotyping strategies. Genes Brain Behav. 2007;6(2):192-200.

2. Wulaer B, Nagai T, Sobue A, Itoh N, Kuroda K, Kaibuchi K, et al. Repetitive and compulsive-like behaviors lead to cognitive dysfunction in Disc1(Delta2-3/Delta2-3) mice. Genes Brain Behav. 2018;17(8):e12478.

3. Wulaer B, Kunisawa K, Hada K, Suento WJ, Kubota H, Iida T, et al. Shati/Nat8l deficiency disrupts adult neurogenesis and causes attentional impairment through dopaminergic neuronal dysfunction in the dentate gyrus. J Neurochem. 2020;10.1111/jnc.15022.

4. Wulaer B, Kunisawa K, Kubota H, Suento WJ, Saito K, Mouri A, et al. Prefrontal cortex, dorsomedial striatum, and dentate gyrus are necessary in the object-based attention test in mice. Mol Brain. 2020;13(1):171.

5. Nagai T, Takuma K, Kamei H, Ito Y, Nakamichi N, Ibi D, et al. Dopamine D1 receptors regulate protein synthesis-dependent long-term recognition memory via extracellular signal-regulated kinase 1/2 in the prefrontal cortex. Learn Mem. 2007;14(3):117-25.

6. Rinaldi A, Mandillo S, Oliverio A, Mele A. D1 and D2 receptor antagonist injections in the prefrontal cortex selectively impair spatial learning in mice. Neuropsychopharmacology. 2007;32(2):309-19.

7. Alkam T, Hiramatsu M, Mamiya T, Aoyama Y, Nitta A, Yamada K, et al. Evaluation of object-based attention in mice. Behav Brain Res. 2011;220(1):185-93.

8. Numa C, Nagai H, Taniguchi M, Nagai M, Shinohara R, Furuyashiki T. Social defeat stress-specific increase in c-Fos expression in the extended amygdala in mice: Involvement of dopamine D1 receptor in the medial prefrontal cortex. Sci Rep. 2019;9(1):16670.
